# Supplementary material for: Thermally-robust spatiotemporal parallel reservoir computing by frequency filtering in frustrated magnets
Source: Sci Rep. 2023 Oct 10;13:15123. doi: 10.1038/s41598-023-41757-3 (PMC10564978; doi:10.1038/s41598-023-41757-3)
Supplement: Supplementary file 1 — Supplementary Information. [file 41598_2023_41757_MOESM1_ESM.pdf]

Supplementary Information for

Thermally-robust spatiotemporal parallel reservoir computing by frequency filtering in frustrated magnets

Kaito Kobayashi and Yukitoshi Motome

*Corresponding author. E-mail: kaito-kobayashi92@g.ecc.u-tokyo.ac.jp

Note 1: Memory capacity task

The memory capacity task [46] serves as a benchmark for evaluating both the STM and nonlinearity of the reservoir. For the n-th order task with the delay d, the target output is defined as $\bar{y}_k = \Pi_{i=0}^{n-1} s'_{k-d-n}$, where s'_k is given by $2s_k - 1$ taking 1 or -1. The output from the reservoir y_k is binarized to $y'_k \in \{1, -1\}$ using a threshold value of 0. The accuracy $p_{n,d}$ is then calculated as the ratio of y'_k matching $\bar{y}_k$. The n-th order memory capacity MC_n is determined based on the mutual information $MI_{n,d}$, and is computed as

$$MI_{n,d} = p_{n,d} \log_2(2p_{n,d}) + (1 - p_{n,d}) \log_2 \left(2(1 - p_{n,d})\right), \qquad MC_n = \sum_{d=0}^{d_{\max}} MI_{n,d}.$$

Figure S1a displays the temperature dependence of MC_n for $n = 1, 2, 3, 4$ with $d_{\max} = 50$. MC_1 decreases as temperature increases, and $MC_{n>1}$ is close to zero, except for MC_3 at $T = 0$, indicating insufficient nonlinearity for this specific task. In Fig. S1b, MC_n is represented when utilizing the spin dynamics within the frequency window $f_{\text{in}} \leq |f| < f_{\text{in}} + 1/(2t_{\text{in}})$. MC_1 shows significant improvement at $T \neq 0$ due to the reduction of thermal noise, while $MC_{n>1}$ still remains close to zero. To achieve better performance for these highly nonlinear tasks with $n > 1$, it would be necessary to introduce a stronger and more complex exchange interaction that is compatible with the linear Zeeman coupling, as discussed in the main text.

Note 2: Echo state property with frequency filtering

The reservoir is considered to possess the echo state property (ESP) when the influence of initial conditions diminishes as time passes. To assess the ESP in our spintronic reservoir, the ESP index [46, 47] was evaluated by giving the same input sequence $\{s_k\}$ via the input magnetic field at frequency f_{in} to multiple distinct initial states with the same energy. The deviation $\delta_k^{1,2}$ between the internal states $\mathbf{X}_k^1$ and $\mathbf{X}_k^2$, obtained from different initial states, is calculated as $\delta_k^{1,2} = 2(\Delta\mathbf{X}_k^1 - \Delta\mathbf{X}_k^2)/(\Delta\mathbf{X}_k^1 + \Delta\mathbf{X}_k^2)$ where $\Delta\mathbf{X}_k^{1,2} = \mathbf{X}_k^{1,2} - \overline{\mathbf{X}}_k^{1,2}$and $\overline{\mathbf{X}}_k^{1,2}$is the mean value of $\mathbf{X}_k^{1,2}$. The ESP index is obtained as $\frac{1}{2000}\sum_{k=1}^{2000}\left(\frac{1}{19}\sum_{i=2}^{20}\delta_k^{1,i}\right)$. If the ESP index is close to zero, the reservoir can be considered to exhibit the ESP.

Figure S2 represents the ESP index while varying the centering frequency of the frequency filter utilized to construct $\{\mathbf{X}_k\}$. At $T = 0$, the ESP index close to zero across all frequencies, indicating the presence of the ESP. Conversely, the ESP index takes nonzero value at $T \neq 0$ because of thermal agitation, which poses an additional challenge to high-performance computation, in addition to the loss of the STM. However, the ESP index approaches zero when utilizing the spin dynamics at around f_{in}, suggesting partial recovery of the ESP by the frequency filter.

Note 3: Thermal robustness for larger input magnetic fields

In Fig. S3, we show the Fourier spectra of the spin dynamics and performance for the STM task when the amplitude of the input magnetic field H_{in} is increased from 0.1, used in the main text, to 0.15 and 0.2. The peak intensity near the frequency f_{in} increases as H_{in} is amplified, greatly exceeding the noise level of thermal fluctuations. Consequently, R^2 around f_{in} is improved, especially for $T = 0.1$, indicating an extension of the operational temperature range.

Note 4: Optimal window of frequency filters

To investigate the information distributions in frequency domain, we simultaneously utilize two frequency windows of $(m_1 - 1)/(2at_{\text{in}}) \leq |f| < m_1/(2at_{\text{in}})$ and $(m_2 - 1)/(2at_{\text{in}}) \leq |f| < m_2/(2at_{\text{in}})$ with positive integers m_1, m_2 and $a = 4, 8$. Figure S4 represents the performance for the STM task of delay $d = 0$ while varying the combination of two frequency windows. R^2 on the diagonal line $m_1 = m_2$, which corresponds to the performance with a single frequency window, is relatively low compared to R^2 with two windows, $m_1 \neq m_2$, demonstrating the performance improvement by utilizing the spin dynamics in a wider frequency range. However, if two frequency windows are mutually linked by the specific unit of $1/t_{\text{in}}$, the performance remains comparable to that obtained with a single window because almost the same information is retained in these frequencies. This is evidenced by the relatively darker lines in Fig. S4 on $m_2/(2at_{\text{in}}) = m_1/(2at_{\text{in}}) \pm n/t_{\text{in}}$ and $m_2/(2at_{\text{in}}) = n/t_{\text{in}} - (m_1 - 1)/(2at_{\text{in}})$ with a positive integer n, which signifies the equivalence of information in f and $f \pm n/t_{\text{in}}$, and f and $n/t_{\text{in}} - f$, respectively. Therefore, the minimum bandwidth required to access all the information is $1/(2t_{\text{in}})$, and the frequency window of $(m - 1)/(2t_{\text{in}}) \leq |f| < m/(2t_{\text{in}})$ adopted in the main text is optimal as it avoids the inclusion of overlapped information.

Note 5: Logic gate operation at different time steps

Beyond the logic gate task with s_k^1 and s_k^2 demonstrated in Fig. 5, the gating of s_{k-d}^1 and s_k^2 can also be computed in our reservoir. Two binary sequences $\{s_k^1\}$ and $\{s_k^2\}$ are simultaneously provided to the input terminal spin at site j via the input magnetic field at frequency $f_{\text{in}}^1 = 8/t_{\text{in}}$ and $f_{\text{in}}^2 = 45/(2t_{\text{in}})$, respectively. The spin dynamics on the neighboring site j^{NN} at zero temperature is utilized for calculation. To incorporate the

information from both input bits, a frequency filter with a window of $f_{\text{in}}^2 - f_{\text{in}}^1 - 1/(t_{\text{in}}) \leq |f| < f_{\text{in}}^2 - f_{\text{in}}^1 + 1/(t_{\text{in}})$ and $f_{\text{in}}^1 + f_{\text{in}}^2 - 1/(t_{\text{in}}) \leq |f| < f_{\text{in}}^1 + f_{\text{in}}^2 + 1/(t_{\text{in}})$ is employed.

Figure S5a displays the reservoir performance R^2, while Fig. S5b illustrates the accuracy for logic gate tasks, including the AND, OR, and XOR tasks, with two input bits at different time steps s_{k-d}^1 and s_k^2. In the case of $d = 0$, the reservoir performs well for all tasks, exhibiting the accuracy close to 1. Nevertheless, as the delay d increases, R^2 gradually diminishes for the AND and OR tasks, while the accuracy converges towards 0.75, which corresponds to the value observed in the random case. In contrast, for the XOR task, R^2 sharply drops to nearly zero at $d = 2$, consequently leading to a decay in accuracy from close to 1 observed for $d = 0$ and 1, to the random case value of 0.5.

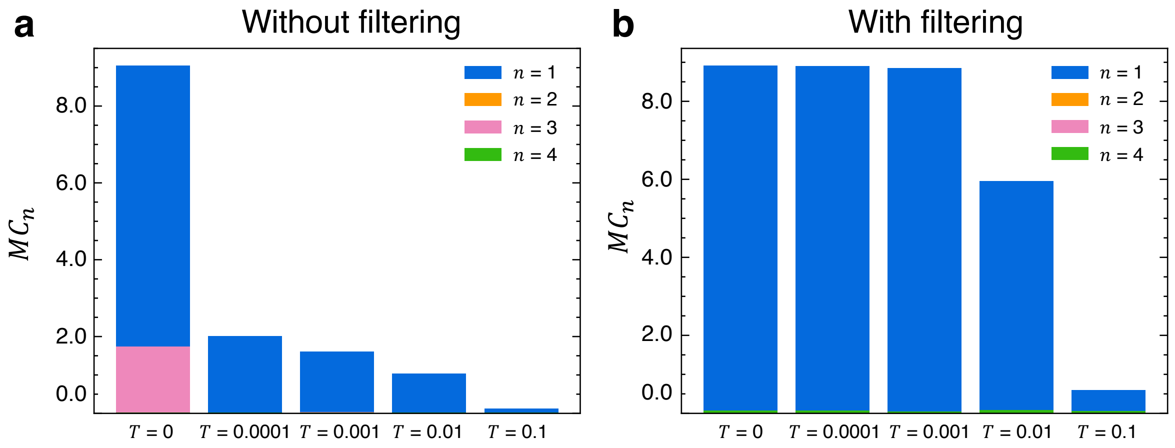

Fig. S1: Memory capacity at finite temperature. a MC_n for $n = 1, 2, 3, 4$ without frequency filtering. Due to thermal noise, $MC_{n=1}$ deteriorates as temperature increases. **b** MC_n with the frequency filter passing $f_{\text{in}} \leq |f| < f_{\text{in}} + 1/(2t_{\text{in}})$, showing significant improvement in $MC_{n=1}$. As for $MC_{n>1}$, the stronger nonlinearity is necessary for better performance.

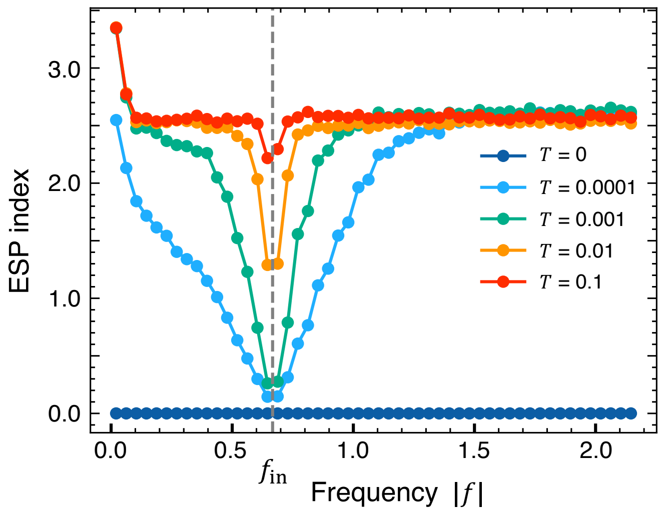

Fig. S2: ESP index with frequency filtering. The ESP index at different temperatures while shifting the center frequency of the frequency filter. The dashed line represents the input frequency f_{in}. At $T = 0$, ESP index is almost zero, but as the temperature increases, it deviates from zero due to thermal agitations. However, when utilizing the frequency filter centering at f_{in}, the ESP index approaches zero even in the presence of thermal noise, demonstrating the partial recovery of ESP.

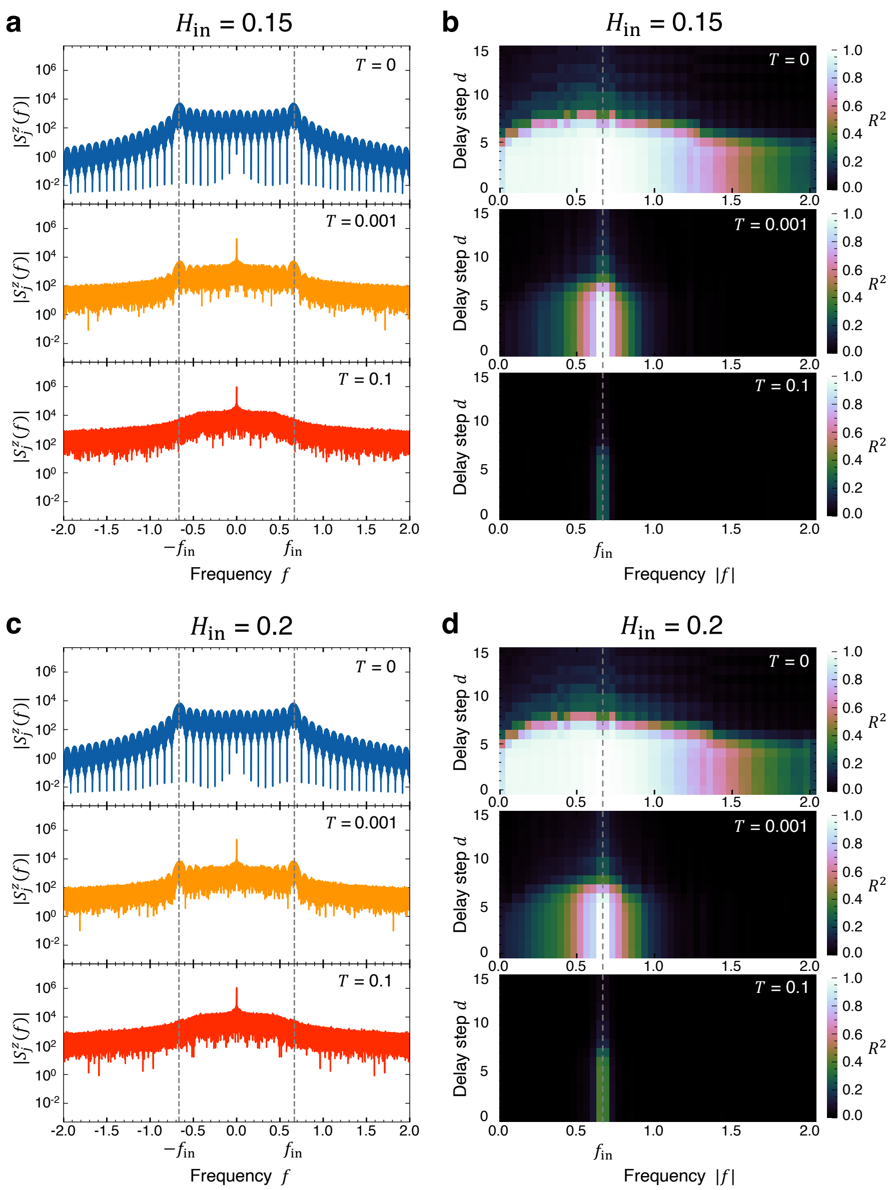

Fig. S3: Reservoir computing at finite temperature with stronger input magnetic fields. a and **c** Fourier spectra of the spin dynamics on the terminal site at three different temperatures. The dashed lines display the input frequency $f_{\mathrm{in}} = 8/t_{\mathrm{in}}$. **b** and **d** Distributions of the STM in frequency domain. The color represents the value of R^2 and the dashed line shows f_{in}. The amplitude of input magnetic field is **a** and **b** $H_{\mathrm{in}} = 0.15$ and **c** and **d** $H_{\mathrm{in}} = 0.2$.

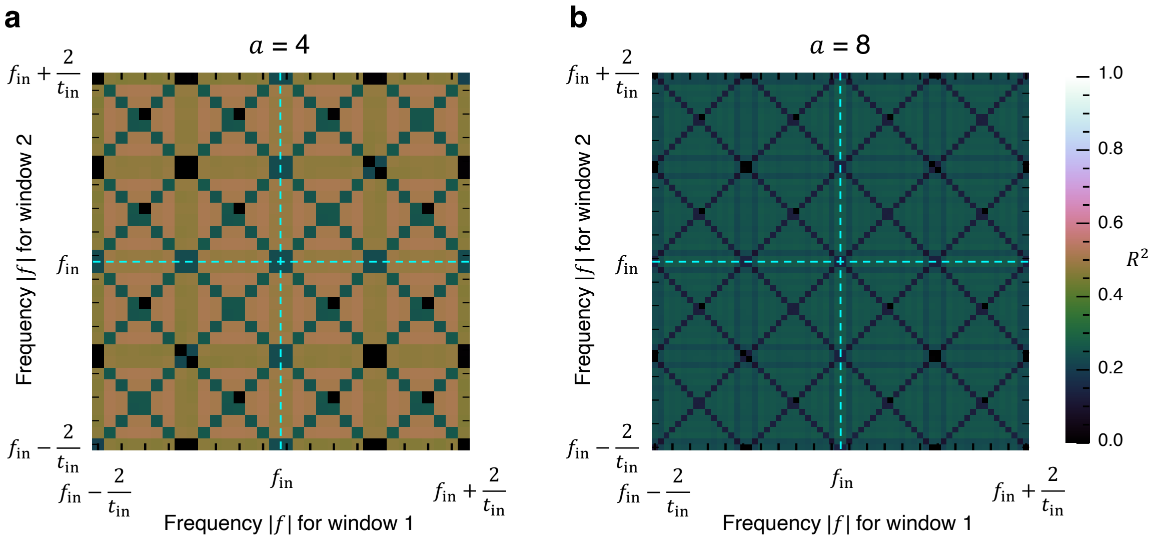

Fig. S4: Performance of the STM task with two frequency windows. **a** and **b** The color plot of R^2 for the STM task of delay step $d = 0$ at zero temperature. The horizontal and vertical axes represent the frequency for the window 1 and 2, respectively. The frequency window 1 passes signals within **a** $(m_1 - 1)/(8t_{\mathrm{in}}) \leq |f| < m_1/(8t_{\mathrm{in}})$, **b** $(m_1 - 1)/(16t_{\mathrm{in}}) \leq |f| < m_1/(16t_{\mathrm{in}})$ with a positive integer m_1. The frequency window 2 similarly transmits signals within the same frequency range with a positive integer m_2 instead.

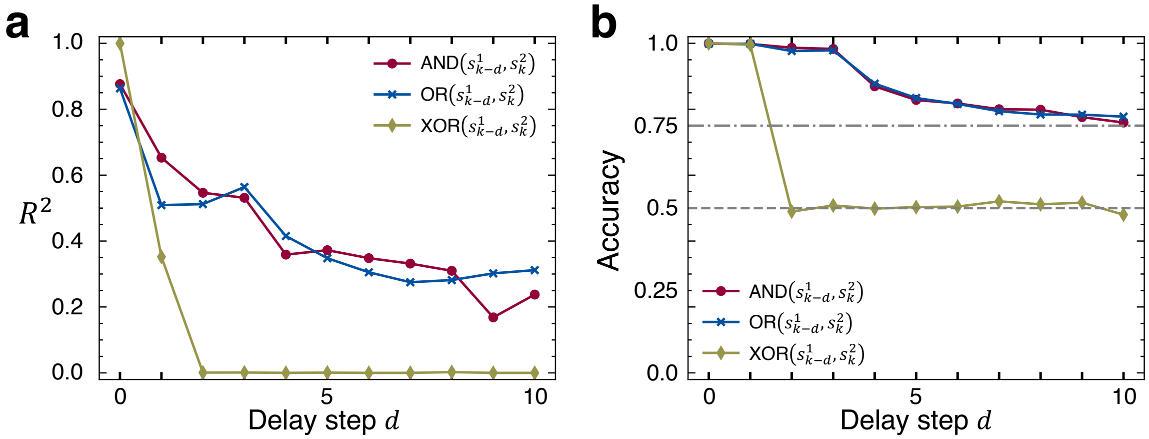

Fig. S5: Performance for the logic gate tasks with two inputs at different time steps. a The reservoir performance R^2 at zero temperature for the AND, OR, and XOR tasks using the inputs s^1_{k-d} and s^2_k, where d denotes the delay step. Frequency filters are applied to the spin dynamics on the neighboring site j^{NN}, specifically targeting the ranges $f^2_{\mathrm{in}} - f^1_{\mathrm{in}} - 1/(t_{\mathrm{in}}) \leq |f| < f^2_{\mathrm{in}} - f^1_{\mathrm{in}} + 1/(t_{\mathrm{in}})$ and $f^1_{\mathrm{in}} + f^2_{\mathrm{in}} - 1/(t_{\mathrm{in}}) \leq |f| < f^1_{\mathrm{in}} + f^2_{\mathrm{in}} + 1/(t_{\mathrm{in}})$. In the case of the AND and OR tasks, R^2 gradually decreases with larger value of d, while for the XOR task, it drops to almost zero at $d = 2$. **b** The accuracy of the predicted answers from the reservoir. For the AND and OR tasks, the accuracy remains close to 1 until $d = 3$, after which it gradually decreases to 0.75, which corresponds to the accuracy in the random case. In contrast, for the XOR task, the accuracy close to 1 for $d = 0$ and 1, but for larger d it drops to the same value of 0.5 as in the random case.
